# Massachusetts Medicaid members that smoked in 2008: Characteristics associated with smoking status in 2014

**DOI:** 10.1371/journal.pone.0186144

**Published:** 2017-10-12

**Authors:** Alexis D. Henry, John Gettens, Judith A. Savageau, Doris Cullen, Anna Landau

**Affiliations:** 1 Center for Health Policy and Research, University of Massachusetts Medical School, Shrewsbury, MA, United States of America; 2 Tobacco Cessation and Prevention Program, Massachusetts Department of Public Health, Boston, MA, United States of America; Legacy, Schroeder Institute for Tobacco Research and Policy Studies, UNITED STATES

## Abstract

The smoking rate among non-elderly Medicaid enrollees is more than double the rate for those privately insured; smoking-related conditions account for 15% of Medicaid expenditures. Under state health reform, Massachusetts Medicaid (MassHealth) made tobacco cessation treatment available beginning in 2006. We used surveys conducted in 2008 and 2014 to examine changes in smoking abstinence rates among MassHealth members identified as smokers and to identify factors associated with being a former smoker. Members previously identified as smokers were surveyed by mail or phone; 2008 and 2014 samples included 3,116 and 2,971 members, respectively. Surveys collected demographic and health information, asked members whether they smoked cigarettes “every day, some days or not at all’, and asked questions to assess smoking intensity among current smokers. The 2014 survey included an open ended-question asking members “what helped the most” in quitting or quit attempts. We observed a significant decrease in members reporting smoking “every/some days” of 15.5 percentage points (p < .0001) from 2008 to 2014, and a significant decrease in smokers reporting smoking “more than 10 cigarettes on days smoked” of 16.7 percentage points (p < .0001). Compared to smokers, former smokers more frequently reported health concerns, the influence of family members, and the use of e-cigarettes as helping the most in quitting. Expanded access to tobacco cessation treatment under the Affordable Care Act may have help to reduce the high smoking rates among Medicaid enrollees. Additionally, smokers’ concerns about health and the influence of family and friends provide opportunities for targeted intervention and messaging about quitting.

## Introduction

Although smoking rates among U.S. adults have declined from 24.7% in 1997 to 15.1% in 2015 [1–2], smoking remains the leading cause of preventable disease and premature deaths for U.S. adults. Despite this decline, smoking rates remain elevated among individuals living in poverty, and those with less education, disabilities, and mental health conditions [2, 3]. Smoking rates are particularly high among Medicaid enrollees with estimated rates of 29.8% among non-elderly Medicaid enrollees compared to 14.0% among the privately insurance [4].

The high rates of smoking among Medicaid enrollees have been found to contribute to increased chronic health conditions and poorer health [5], as well as high smoking-related expenditures [6]. A recent study estimated 2010 Medicaid expenditures attributable to smoking at over $39 billion, accounting for 15% of Medicaid’s expenditures [6]. Given the health consequences and high costs associated with smoking, efforts to reduce smoking should be a critical concern for Medicaid policy makers. In 2006, under state healthcare reform, Massachusetts Medicaid (MassHealth) began to offer tobacco cessation treatments (TCT) benefits, including all FDA-approved medications and counseling services. Initially a pilot, the benefit was made permanent in 2008 [7]. Subsequently, in January 2014 the Affordable Care Act (ACA) required state Medicaid programs to cover tobacco cessation medications except for counseling. However, 43 states, including Massachusetts, also cover counseling [8].

Approximately 68.8% of current smokers want to stop smoking and 52.4% had a past year quit attempt [9, 10]. Studies examining Medicaid coverage for TCT prior to the ACA found coverage to be associated with increased likelihood of quit attempts and/or successful quits [11, 12]. Little is known, however, about changes in smoking abstinence rates over time, and factors associated with former smoking in a Medicaid program with TCT benefits. Previously, we found benefit awareness was high among MassHealth members who smoked [13], likely due to active statewide publicity efforts [7]. Herein, we report 2008 and 2014 survey findings of MassHealth members identified as smokers in 2007. The two surveys allowed us to examine changes in smoking abstinence rates over time, and to identify factors associated with being a former smoker.

## Methods

### Survey procedure

Our study used data from the 2008 (S1 File) and 2014 (S2 File) MassHealth Smoking Cessation (MHSC) Surveys. We obtained approval for both surveys from the University of Massachusetts Medical School’s Institutional Review Board (IRB). For both surveys, the IRB approved a waiver of written documentation of consent. (The consent processes used are described further below). We conducted the surveys in both English and Spanish with non-elderly adult (age 18 to 64) MassHealth members who were identified as having a recent smoking history as of January 2008, defined in one of two ways.: First, using MassHealth claims data, members were identified that one or more claims for TCTs in September 2007. Because of TCT use, we assumed the members were either a current smoker or recent smoker as of September 2007. Second, members were identified if they did not have TCT claims between July 1, 2006 and September 30, 2007 and they responded ‘yes’ to a single-question survey asking “Have you smoked any cigarettes in the last six months?” administered in November 2007. (The single-question survey was also approved by the UMMS IRB). Using these two approaches the sample represented MassHealth members with a recent smoking history as of January 2008., The 2008 sample included 3,116 MassHealth members, and the 2014 sample included 2,971 MassHealth members. The population sampled in 2014 was a subset of the population sampled in 2008 because some people in the 2008 population were no longer MassHealth members in 2014.

We used a dual-mode (mail and phone) method to administer both surveys, which included two mailings of the survey (the second mailed approximately two weeks after the first) and six attempts to complete telephone interviews of initial non-responders. The mailed surveys were in English; telephone interviews were conducted in English and Spanish. The mailed survey packet included a study fact sheet describing the purpose of the survey and ensuring members of the confidentiality of their responses; members responding by mail were presumed to consent to the survey. Prior to conducting interviews with members contacted by phone the interviewers described the purpose of the survey, assured members of the confidentiality of their responses and obtained members’ verbal consent. The 2008 survey was administered between January and March 2008, and the 2014 survey was administered between April and July 2014.

Both surveys included questions regarding the following: current and prior smoking behavior; attempts to quit smoking; chronic health conditions and/or mental illness diagnoses; and demographic characteristics. We used responses to the question, “Do you now smoke cigarettes every day, some days, or not at all?” to determine smoking status. The three response categories (every day, some days, not at all) were used to determine smoking status, either as a smoker (i.e., smokes some days or every day) or former smoker (i.e., smokes not at all). The following three questions were used to determine three separate smoking intensity measures:

1. What is the number of days you smoked in the last 30 days? (open text)
2. On days that you smoke, how many cigarettes do you usually smoke a day? (open text)
3. On days that you smoke, how soon after you wake up do you usually smoke your first cigarette of the day? (within 5 minutes; 6 to 30 minutes; 31 to 60 minutes; or more than 60 minutes)

The responses were dichotomized to estimate the following: the percent smoking more than 21 cigarettes in the past 30 days; the percent smoking more than ten cigarettes per day; and/ the percent smoking within five minutes of waking.

In addition, the 2014 survey included an open-ended question asking members to identify “what helped you the most” in their quit attempts (smokers) or their most recent quit (former smokers). We post-coded responses about helpful strategies into the following 12 categories (informed by the literature): avoiding smokers or smoking places; medication (Chantix, Zyban or Welbutrin); nicotine replacement therapy; e-cigarettes; cost; determination; distractions; family members’ influence; health concerns; pregnancy; nothing was helpful; and other. Members were also asked whether they had used electronic cigarettes (e-cigarettes) in the past 30 days.

### Data analysis

We used SAS (Version 9.3) for all data analysis. Survey responses were weighted to reflect the probability of selection. We generated descriptive statistics for all responses, and calculated smoking status (smoker vs. former smoker) and smoking intensity (number of days smoked; number of cigarettes smoked; time of first cigarette) percentages for 2008 and 2014, as well as the differences in percentages between the two cohorts. We used Generalized Estimating Equations (Proc Genmod) to test the statistical significance of these differences, accounting for non-independent observations and survey design. We adjusted these estimates to account for differences in the characteristics of the two samples, adjusting for self-reported age, gender, education, ethnicity, race, chronic health conditions (asthma, COPD/emphysema, lung cancer, colon cancer, hypertension, heart disease, diabetes), and mental illness.

We subsequently used logistic regression (Proc Survey Logistic) to determine the characteristics associated with smoking status among the 2014 survey respondents as the dependent variable. We generated odds ratios for each member characteristic; statistical significance was established at p < .05 (95% confidence intervals). The 2014 member characteristics entered as independent variables included:

- Demographic characteristics–age, gender, education level, employment status, ethnicity, race, language spoken at home;
- Health conditions/healthcare visit–asthma, COPD/emphysema, hypertension, heart disease, diabetes, mental illness, a healthcare visit in the past 12 months;
- Social factors–lives with smokers, has friends that smoke;
- Used electronic cigarette in the past 30 days; and
- Factors that ‘helped the most’ in a quit or quit attempt.

## Results

Survey Response rates for 2008 and 2014 were 52% (n = 1,635) and 27% (n = 793), respectively. Demographic characteristics and health conditions of respondents are presented in Table 1.

**Table 1:** Demographic characteristics of MassHealth members responding to the 2008 & 2014 MassHealth smoking cessation surveys.

	Survey Year	
Characteristics	2008 weighted % (n)^a^	2014 weighted % (n)
**Age**		
18 to 24	7.4% (107)	—
25 to 34	15.9% (259)	10.9% (75)
35 to 44	22.3% (381)	13.2% (121)
45 to 54	31.4% (510)	32.7% (243)
55 to 64	21.5% (320)	33.1% (226)
65 or older	1.4% (21)	10.2% (69)
**Gender**		
Female	58.6% (1046)	63.3% (500)
Male	41.5% (555)	36.7% (233)
**Level of Education**		
8^th^ grade or less	8.1% (122)	5.6% (33)
Some high school or less	14.4% (252)	12.9% (89)
High school graduate or completed GED	39.5% (646)	36.2% (291)
Some college or 2 year degree	30.9% (485)	36.2% (250)
4 year degree	4.6% (60)	7.1% (41)
More than 4-year college degree	2.6% (28)	2.0% (15)
**Employment status**		
Currently working	N/A	21.7% (171)
Not working	N/A	78.3% (561)
**Ethnicity**		
Hispanic	15.1% (199)	13.3% (78)
Non-Hispanic	84.9% (1363)	86.7% (647)
**Race**		
Non-white	20.3% (274)	21.2% (120)
White	79.7% (1284)	78.8% (598)
**Language spoken at home**		
Non-English	12.4% (157)	7.9% (69)
English	87.7% (1426)	92.1% (677)
**Chronic Health Conditions**		
Asthma	27.8% (499)	33.4% (242)
Emphysema or COPD	13.3% (263)	28.1% (208)
Hypertension	27.1% (449)	43.3% (295)
Heart disease	7.6% (125)	9.6% (77)
Diabetes	12.6% (207)	22.8% (153)
Mental Health Condition	61.5% (1055)	68.8% (531)

Abbreviations: GED, general educational development; N/A, not asked; COPD, chronic obstructive pulmonary disease

^a^N’s are non-weighted.

From 2008 to 2014, the percent of respondents that reported smoking every day or some days dropped 15.6 percentage points (p < .0001) from 88.2% to 72.4% (Table 2). Similarly, the percent of smokers that ‘smoked more than 10 cigarettes on days smoked’ dropped 16.7 percentage points (p < .0001) from 2008 to 2014. However, not statistically significant were differences in the percent of smokers that ‘smoked more than 21 days in the last 30 days’ (a decrease of 2.0 percentage points) or the percent of smokers that ‘smoked within 5 minutes after waking’ (an increase of 4.1 percentage points).

**Table 2:** Smoking status and smoking intensity of 2,013 MassHealth members, MassHealth smoking cessation survey data, 2008 and 2014.

	2008 Survey, weighted % (n)	2014 Survey, weighted % (n)	Point Difference (P Value)^a^	Adjusted Point Difference (P Value)^b^
Smokes every day or some days^c^	88.2% (1,343)	72.4% (519)	-15.8 (.0001)	-15.5 (.0001)
Smokes more than 21 days in last 30 days^d^	85.8% (1,079)	84.5% (432)	-1.3 (.59)	-2.0 (.48)
Smokes more than 10 cigarettes on days smoked^d^	56.5% (736)	45.9% (251)	-10.4 (.0023)	-16.7 (.0001)
First smokes within 5 minutes of waking^d^	26.0% (365)	30.0% (172)	4.0 (.17)	4.1 (.23)

^a^The differences were estimated using Generalized Estimating Equations (GEE) to account for non-independent observations.

^b^The differences were estimated using GEE. The differences were adjusted for self-reported age, gender, education, ethnicity, race, chronic condition status (asthma, emphysema or COPD, lung cancer, colon cancer, hypertension, heart disease, diabetes), and mental illness.

^c^Statistics based on 1,585 2008 Survey responses and 743 2014 Survey responses.

^d^Statistics based on respondents that smoke every day or some days.

Table 3 provides descriptive statistics (frequencies) for smokers and former smokers. Results of the logistic regression (Table 3) showed no statistically significant association between member demographic characteristics (age, gender, education, employment status, ethnicity, race and language) and smoking status. Among chronic health conditions, self-reported diabetes was significantly associated with an increased odds of being a former smoker (OR = 2.7; 95% CI = 1.0–7.6), while COPD/emphysema was associated with a decreased odds of being a former smoker (OR = 0.3, 95% CI = 0.1–0.9). There were no other significant associations between health conditions or healthcare utilization and smoking status.

**Table 3:** Comparison of smokers and former smokers among 677 MassHealth members, MassHealth smoking cessation survey data, 2014.

Characteristics	Frequencies		Logistic Regression^a^
	Smokers weighted % (n)	Former Smokers weighted % (n)	Odds Ratio (95% CI)
**Age**			
25 to 34	9.5% (45)	15.9% (24)	3.5 (0.6. 19.3)
35 to 44	12.7% (81)	13.2% (30)	0.6 (0.1, 3.5)
45 to 54	37.0% (157)	23.2% (61)	0.6 (0.1. 2.8)
55 to 64	31.4% (132)	36.8% (74)	1.6 (0.4, 7.5)
65 or older	9.5% (34)	11.0% (30)	Reference
**Gender**			
Female	63.7% (306)	64.0% (152)	1.3 (0.4, 7.5)
Male	36.3% (143)	36.0% (67)	Reference
**Level of Education**			
Some high school or less	21.0% (78)	14.2% (27)	1.2 (0.2, 7.0)
High school graduate or GED	35.2% (175)	32.9% (86)	1.9 (0.5, 6.8)
Some college or 2 year degree	35.1% (151)	43.0% (86)	1.8 (0.5, 6.5)
4 year degree or more	8.7% (33)	9.9% (19)	Reference
**Employment status**			
Currently working	21.2% (102)	24.0% (58)	1.3 (0.6, 3.0)
Not working	78.8% (347)	76.0% (161)	Reference
**Ethnicity**			
Hispanic	15.6% (55)	12.0% (19)	1.1 (0.3, 3.9)
Non-Hispanic	84.4% (388)	88.0% (199)	Reference
**Race**			
Non-white	22.2% (83)	21.4% (31)	1.0 (0.3. 3.3)
White	77.8% (356)	78.6% (185)	Reference
**Language spoken at home**			
Non-English	8.6% (32)	8.4% (15)	0.8 (0.1, 3.7)
English	91.4% (413)	91.6% (202)	Reference
**Chronic Conditions**			
Asthma	34.2% (152)	32.4% (72)	0.6 (0.3, 1.5)
COPD/Emphysema	**29.5% (129)**	**24.2% (58)**	**0.3 (0.1, 0.9)**
Hypertension	43.8% (181)	40.6% (89)	0.7 (0.3, 1.6)
Heart disease	9.3% (48)	12.7% (24)	0.4 (0.1, 1.7)
Diabetes	**20.8% (85)**	**26.7% (54)**	**2.7 (1.0, 7.6)**
**Mental illness**			
Yes	72.3% (344)	65.2% (147)	0.7 (0.3, 1.7)
No	27.7% (107)	34.8% (68)	Reference
**Healthcare visit in past 12 months**			
Yes	89.4% (402)	93.1% (203)	1.7 (0.5, 6.0)
No	10.6% (47)	7.0% (12)	Reference
**E-cigarette use in past 30 days**			
Yes	**23.5% (114)**	**10.0% (21)**	**0.1 (0.02, 0.2)**
No	76.5% (333)	90.0% (198)	Reference
**Living with smokers**			
Yes	44.7% (202)	22.1% (51)	Reference
No	55.3% (247)	77.9% (167)	2.0 (0.9. 4.3)
**Has friends that smoke**			
Yes	89.4% (414)	80.9% (176)	Reference
No	10.6% (36)	19.1% (41)	2.3 (0.8, 6.4)
**Helped most in quit or quit attempts**			
Avoiding smokers/smoking places	7.1% (23)	0.3% (3)	0.2 (0.1, 1.5)
Chantix, Zyban, Welbutrin	17.4% (108)	10.4% (43)	2.3 (0.6, 8.4)
Cost of cigarettes	2.5% (6)	6.7% (9)	1.3 (0.2, 8.3)
Determination	24.4% (75)	24.5% (46)	2.5 (0.7, 9.0)
Distractions	9.1% (40)	3.4% (10)	1.0 (0.2, 4.9)
E-cigarettes	**1.3% (15)**	**12.6% (21)**	**312 (48, 999)**
Family members’ influence	**4.2% (19)**	**19.4% (31)**	**8.4 (2.3, 31.2)**
Health concerns	**5.0% (24)**	**27.9% (56)**	**13.3 (3.1, 57.8)**
Nicotine replacement therapy	17.3% (85)	6.2% (15)	0.5 (0.1, 2.1)
Nothing	**9.7% (32)**	**1.5% (3)**	**0.04 (0.01, 0.5)**
Pregnancy	2.6% (8)	0.2% (2)	0.1 (0.01, 1.02)
Other	12.1% (47)	6.4% (17)	1.1 (0.2, 6.6)

Abbreviations: CI, confidence intervals; GED, general educational development; COPD, chronic obstructive pulmonary disease.

^a^The dependent variable is smoking status (former smoker = 1; smoker = 0).

The use of e-cigarettes within the last 30 days was significantly associated with a decreased odds of being a former smoker (OR = 0.1, 95% CI = 0.02–0.2). Among the factors that smokers identified as ‘helped the most’ in quitting/quit attempts, three were significantly associated with an increased odds of being a former smoker: health concerns (OR = 13.3, 95% CI = 3.1–57.8); influence of family members (OR = 8.4, 95% CI = 2.3–31.2); and using e-cigarettes (OR = 312, 95% CI = 48–999). Approximately 27.9% of former smokers reported that ‘health concerns’ helped them to quit compared to 5.0% smokers. Comparably, 19.4% of former smokers reported ‘family members’ influence’ compared to 4.2% of smokers. Only 1.5% of smokers reported that e-cigarettes were helpful compared to 12.6% of former smokers. As expected a higher percentage of smokers reported that ‘nothing’ helped compared to former smokers (10% vs. 2%) and this was associated with a decreased odds of being a former smoker (OR = 0.04, 95% CI = 0.0–0.5).

## Discussion

Estimated smoking rates among adult Medicaid enrollees are more than double those privately insured [4]. Identifying successful approaches to reducing smoking is an important concern for healthcare providers, Medicaid policy makers, and public health officials. In their recent article, Ku and colleagues [14] find that the use of Medicaid cessation benefits among enrollees varies widely across states and suggest that most states could do much more to achieve higher rates of use. Controlling for demographic and health characteristics, we observed a significant reduction in smoking rates between 2008 and 2014 among MassHealth members identified as having a recent smoking history in late 2007. Although we cannot attribute this decline specifically to MassHealth’s tobacco cessation benefit availability, this finding is encouraging. Still, the overwhelming majority of surveyed members that reported smoking in 2008 were still smoking in 2014 despite the availability of the benefit suggests that access to cessation benefits alone is not sufficient to help many people quit. Data from the 2013 Behavioral Risk Factor Surveillance System indicated that two-thirds of adult smokers had made quit attempts in the past year [9]. However, relapse rates are high and may explain, in part, the reason why the majority of members still smoked in 2014 [15, 16].We also observed a significant decrease over time in the percentage reporting smoking more than ten cigarettes per day. However, we did not observe significant differences in the percentage reporting smoking at least 21 days in the past month. Smokers may find it easier to reduce the number of cigarettes they smoke daily than to not smoke in a day. Additionally, there is some evidence that smoke-free policies can result in reduced cigarette consumption among those who continue to smoke [17], and Massachusetts has some of the strongest smoke-free laws nationally [18]. We also did not observe significant differences in the percentage reporting smoking their first cigarette within five minutes of waking. Smoking shortly after waking is considered an important predictor of nicotine dependence, and it may be particularly difficult for smokers to modify this behavior [19].

Using 2014 survey data, we found no significant associations between smoking status and demographic characteristics, healthcare utilization, and most of the health conditions queried. However, we did observe significant associations between smoking status and self-reported COPD/emphysema and diabetes. Members reporting COPD/emphysema were significantly less likely, while those reporting diabetes were significantly more likely, to be former smokers. The link between COPD and cigarette smoking is well-established. Smoking is considered the primary preventable risk factor for the development of COPD [20]. Conversely, prevalence of smoking among adults with diabetes is comparable or somewhat lower than among the general population [21]. While a link between smoking and the development of diabetes is less well-established, the 2014 Surgeon General’s Report on smoking noted that the risk of developing diabetes is 30–40% higher for active smokers than non-smokers [20]. Smoking cessation treatments are critical in the management of chronic diseases such as COPD and diabetes [22]. Studies comparing the effectiveness of smoking cessation treatment for different chronic conditions are lacking; those with COPD may have a higher level of tobacco addiction and more difficulty quitting than those with other chronic conditions.

Of the factors identified by MassHealth members as ‘helping the most’ in quitting or quit attempts, three factors differed between smokers and former smokers: health concerns; influence of family members; and use of electronic cigarettes. Former smokers were significantly more likely to report each of these as helpful compared to smokers. Concerns about health are consistently found to be primary factors motivating smokers to quit. A 2006 review of 30 studies examining smokers’ reasons for quitting found health consequences of smoking to be the most consistent motivation [23]. A 2014 qualitative study corroborated findings that concerns about health were one of the most important motivations to quit [24].

Consistent with our findings of family member’s influence, prior studies have noted the importance of social influences in smoking cessation, such as family members urging the smoker to quit or informal bans on smoking at home [24, 25]. Christakis and Fowler [26] applied a network analysis approach to examine social influences on smoking behavior among a Framingham Heart Study cohort (from 1971 to 2003). Smoking cessation by a spouse, sibling or a friend were all significantly associated with a decreased risk of smoking. They also observed relatively separate “clusters” of smokers and non-smokers, and posit that the person-to-person spread of smoking cessation is a factor in “the population-level declines in smoking seen in recent decades” (p. 2256). A recent analysis of U.S. Behavioral Risk Factor Surveillance System data (1996 to 2012) showed that declines in smoking were greater in more in affluent versus poorer communities [27]. Because they have lower income, Medicaid enrollees may live in areas with more smokers and have less exposure to the social influences supporting quitting.

E-cigarettes are becoming increasingly popular. Estimates suggest that about half of current or recent former smokers in the U.S. have tried e-cigarettes [28]. Use of e-cigarette in Medicaid populations has not been widely studied; however, our finding that almost one-quarter of smokers had used e-cigarettes within the past 30 days suggests that use of these devices may be a growing trend among Medicaid enrollees. Former smokers were significantly more likely than smokers to identify e-cigarettes as that which ‘helped the most’ in quitting or quit attempts. We were not able to determine if substitution of e-cigarettes for tobacco cigarettes played a direct role in the reduced number of cigarettes smoked per day among smokers and in the overall reduced smoking rates reported from 2008 to 2014. Studies show that e-cigarette use as a cessation strategy among smokers is on the rise [29] though the efficacy of e-cigarettes in smoking cessation is unclear as is their long-term safety [30].

Massachusetts was one of the first states nationally to offer comprehensive tobacco cessation benefits to Medicaid enrollees, beginning in 2006. Although we cannot directly attribute the observed decrease over the six-year period in the smoking rate among adult smokers to the availability or use of the benefit, this finding is promising. At more than double the national average, the high smoking rates among Medicaid enrollees, along with smoking-related disease costs, present significant challenges to Medicaid agencies, healthcare providers, and public health officials. Although tobacco cessation medication benefits are now covered by Medicaid in every state, recent data shows that only 10% of current Medicaid smokers use these benefits [14]. Benefit use varies widely across states; states that have expanded Medicaid under ACA show much higher utilization of medication benefits than non-expansion states. Coordinated efforts across Medicaid and public health agencies are needed to increase awareness and utilization of these benefits, as are studies that identify effective strategies to support the use of TCT and quitting among Medicaid patients [14].

There are a number of limitations to this study worth noting. Self-reported survey responses are subject to information bias in general with a specific possibility that social desirability might have resulted in over-reporting a reduction in, or quitting, smoking. We had a lower response rate in the 2014 cohort, and low response numbers to some key variables included in the logistic regression. Medicaid populations are often transient and difficult to reach; this likely contributed to the lower response rate in 2014 given the six-year time span between surveys. The possibility of a non-response bias also exists; it is possible that former smokers were more likely to respond to the survey compared to smokers. If this is the case, the reduction in the percentage smoking in 2014 would be over-estimated. Finally, this study was conducted in one state which might limit the generalizability of findings, given the statewide healthcare reform activities in Massachusetts preceding national healthcare reform. Despite these limitations, strengths of the study include the comparison of two Medicaid population cohorts over time and a sufficiently large sample to assess changes in smoking status and factors associated with being a former smoker.

## Conclusions

We used surveys conducted in 2008 and 2014 to examine changes in smoking rates among MassHealth members identified as smokers and to identify factors associated with being a former smoker. We observed a substantial reduction in the smoking rate; however, the overwhelming majority of surveyed members that reported smoking in 2008 were still smoking in 2014. Our findings suggest that health and social factors are important contributors to successful cessation. Also, it appears that some smokers are using e-cigarettes to reduce or quit smoking. This finding is unexpected and additional research is needed to understand this relationship.

Smokers’ health concerns and the influence of family and friends provide an opportunity for targeted interventions and messaging. Additional research on the role of social networks to successful quitting may inform both the development of new counseling approaches as well as the knowledge gaps in smoking cessation education.

## Supporting information

S1 File 2008 MassHealth smoking cessation survey. (PDF)

S2 File 2014 MassHealth smoking cessation survey. (PDF)

S3 File SAS data set Supporting Table 1 and Table 2 analysis. (SAS7BDAT)

S4 File SAS data set Supporting Table 3 analysis. (SAS7BDAT)

S5 File SAS format file. (SAS)

## References

[1] U.S. Department of Health and Human Services, Centers for Disease Control and Prevention, National Center for Health Statistics. Early release of selected estimates based on data from the National Health Interview Survey, 2015. Released 05/16. http://www.cdc.gov/nchs/data/nhis/earlyrelease/earlyrelease201605_08.pdf. Accessed October 5, 2016.

[2] Jamal A, Homa DM, O’Connor E, Babb SD, Caraballo RS, Singh T, et al Current cigarette smoking among adults–United States, 2005–2014. MMWR Morb Mortal Wkly Rep 2015;64(44):1233–40. doi: 10.15585/mmwr.mm6444a2 26562061 10.15585/mmwr.mm6444a2

[3] Goren A, Annunziata K, Schnoll RA, Suaya JA. Smoking cessation and attempted cessation among adults in the United States. PLoS One 2014; 9(3):e93014 doi: 10.1371/journal.pone.0093014 24676348 10.1371/journal.pone.0093014 PMC3968049

[4] US Department of Health and Human Services, Centers for Disease Control and Prevention, National Center for Health Statistics. Summary health statistics: National Health Interview Survey, 2014. Table A-12a 2014. http://ftp.cdc.gov/pub/Health_Statistics/NCHS/NHIS/SHS/2014_SHS_Table_A-12.pdf. Accessed on June 3, 2016.

[5] Freeman R, Lybecker KM, Taylor WD. The effectiveness of disease management programs in the Medicaid population Hamilton (ON): Cameron Institute; 2011.

[6] Xu X, Bishop EE, Kennedy SM, Simpson SA, Pechacek TF. Annual healthcare spending attributable to cigarette smoking: An update. Am J Prev Med 2015;48(3):326–33. doi: 10.1016/j.amepre.2014.10.012 25498551 10.1016/j.amepre.2014.10.012 PMC4603661

[7] Massachusetts Department of Public Health. MassHealth Smoking Cessation Benefit: Briefing Notes. MA DPH Tobacco Cessation and Prevention Program, Updated 5/24/2012. http://www.mass.gov/eohhs/docs/dph/tobacco-control/masshealth-smoke-cessation-benefit.pdf. Accessed on January 18, 2016.

[8] Henry J. Kaiser Family Foundation. State Medicaid Program Coverage of Tobacco Dependence Treatments by Type of Coverage. http://kff.org/medi/state-indicator/cessation-treatment-under-medicaid/. Accessed on January 18, 2016.

[9] Lavinghouze SR, Malarcher A, Jama A, Neff L, Debrot K, Whalen L. Trends in quit attempts among adult cigarette smokers–United States, 2001–2013. MMWR Morb Mortal Wkly Rep 2015; 64(40):1129–35. doi: 10.15585/mmwr.mm6440a1 26468619 10.15585/mmwr.mm6440a1

[10] Centers for Disease Control and Prevention. Quitting smoking among adults–United States, 2001–2010. MMWR Morb Mortal Wkly Rep 2011;60(44):1513–19. 22071589

[11] Greene J, Sacks RM, McMenamin SB. The impact of tobacco dependence treatment coverage and copayments in Medicaid. Am J Prev Med 2014;46(4):331–6. doi: 10.1016/j.amepre.2013.11.019 24650835 10.1016/j.amepre.2013.11.019

[12] Liu F. Quit attempts and intention to quit cigarette smoking among Medicaid recipients in the USA. Public Health 2010;124:553–8. doi: 10.1016/j.puhe.2010.05.015 20832833 10.1016/j.puhe.2010.05.015

[13] Gettens J, Savageau JA, Mitra M, Henry A, Keithly L, Paskowsky M. Tobacco cessation and chronic conditions in the Massachusetts Medicaid program. Center for Health Policy and Research, University of Massachusetts Medical School, Shrewsbury MA; 2013.

[14] Ku L, Bruen BK, Steinmetz K, Bysshe T. Medicaid tobacco cessation: Big gaps remain in efforts to get smokers to quit. Health Aff 2016; 35(1):62–70. 10.1377/hlthaff.2015.0756 PMC4705858 26733702

[15] World Lung Foundation. The Tobacco Atlas, 5^th^ edition Atlanta (GA): American Cancer Society; 2015.

[16] Prochaska JJ. Nicotine replacement therapy as a maintenance treatment. JAMA 2015;314(7):718–9. doi: 10.1001/jama.2015.7460 26284723 10.1001/jama.2015.7460 PMC5131795

[17] Berg CJ, Haardofer R, Windle M, Solomon M, Kegler MC. Smoke-free policies in multiunit housing: Smoking behavior and reactions to messaging strategies in support or in opposition. Prev Chronic Dis 2015;12 (E98). http://www.cdc.gov/pcd/issues/2015/14_0479.htm. Accessed June 4, 2016. 10.5888/pcd12.140479 PMC4492220 26111158

[18] American Lung Association. State of tobacco control: Smokefree air laws. http://www.lung.org/our-initiatives/tobacco/reports-resources/sotc/state-grades/state-rankings/smokefree-air-laws.html. Accessed June 3, 2016.

[19] Heatherton TF, Kozlowski LT, Frecker RC, Fagerström KO. The Fagerström test for nicotine dependence: A revision of the Fagerström Tolerance Questionnaire. Br J Addict 1991;86:1119–27. 1932883 10.1111/j.1360-0443.1991.tb01879.x

[20] U.S. Department of Health and Human Services, Public Health Service, Office of the Surgeon General. The health consequences of smoking—50 years of progress: A report of the Surgeon General, 2014. http://www.surgeongeneral.gov/library/reports/50-years-of-progress/index.html#fullreport. Accessed on July 7, 2016.

[21] Fan AZ, Rock V, Zhang X, Li Y, Elam-Evans L, Balluz L. Trends in cigarette smoking rates and quit attempts among adults with and without diagnosed diabetes, United States, 2001–2010. Prev Chronic Dis 2013;10:120259 http://dx.doi.org/10.5888/pcd10.120259. Accessed on January 4, 2016. 10.5888/pcd10.120259 PMC3780710 24050530

[22] Gritz ER, Vidrine DJ, Fingeret MC. Smoking cessation: A critical component of medical management in chronic disease populations. Am J Prev Med 2007;33(6S):S414–22. 18021917 10.1016/j.amepre.2007.09.013

[23] McCaul KD, Hockemeyer JR, Johnson RJ, Zetocha K, Quinlan K, Glasgow RE. Motivation to quit using cigarettes: A review. Addict Behav 2006;31:42–56. doi: 10.1016/j.addbeh.2005.04.004 15916861 10.1016/j.addbeh.2005.04.004

[24] Buczkowski K, Marcinowicz L, Czachowski S, Piszczek E. Motivations toward smoking cessation, reasons for relapse, and modes of quitting: Results from a qualitative study among former and current smokers. Patient Prefer Adherence 2014;4(8):1353–63. 10.2147/PPA.S67767 PMC4199752 25336926

[25] Zablocki RW, Edland SD, Myers MG, Strong DR, Hofstetter CR, Al-Delaimy WK. Smoking ban policies and their influence on smoking behaviors among current California smokers: A population-based study. Prev Med 2014;59:73–8. doi: 10.1016/j.ypmed.2013.11.018 24291748 10.1016/j.ypmed.2013.11.018

[26] Christakis NA, Fowler JH. The collective dynamics of smoking in a large social network. N Engl J Med 2008;358(21):2249–58. doi: 10.1056/NEJMsa0706154 18499567 10.1056/NEJMsa0706154 PMC2822344

[27] Dwyer-Lindgren L, Mokdad AH, Srebotnjak T, Flaxman AD, Hansen GM, Murray CJL. Cigarette smoking prevalence in US counties: 1996–2012. Pop Health Metr 2014;12(5):1–13. 10.1186/1478-7954-12-5 PMC3987818 24661401

[28] Schoenborn CA, Gindi RM. Electronic cigarette use among adults: United States, 2014. NCHS Data Brief 2015;217:1–8. 26555932

[29] Pokhrel P, Herzog TA. Reasons for quitting cigarette smoking and electronic cigarette use for cessation help. Psychol Addict Behav 2015;29(1):114–21. doi: 10.1037/adb0000025 25180551 10.1037/adb0000025 PMC4511704

[30] Orr KK, Asal NJ. Efficacy of electronic cigarettes for smoking cessation. Ann Pharmacother 2014;48(11):1502–6. doi: 10.1177/1060028014547076 25136064 10.1177/1060028014547076

