# Supplementary material for: Massachusetts Medicaid members that smoked in 2008: Characteristics associated with smoking status in 2014
Source: PLoS One. 2017 Oct 12;12(10):e0186144. doi: 10.1371/journal.pone.0186144 (PMC5638442; doi:10.1371/journal.pone.0186144)
Supplement: S1 File — (PDF) [file pone.0186144.s001.pdf]

MassHealth

Smoking Cessation Benefit Survey

Please fill out this survey thinking about your own health and health care.

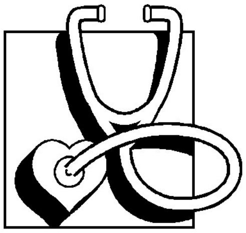

Your answers are important whether you smoke or not. We want to know your experience with MassHealth benefits that help people to quit smoking.

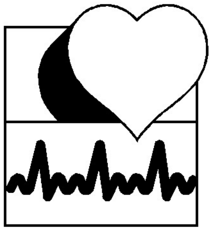

Please take a few minutes to fill out this survey. Return it to us in the envelope provided which already has an address and stamps.

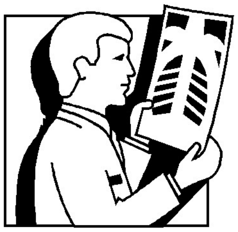

It is your decision whether or not to complete this survey. Your decision will not affect your MassHealth benefits. Your answers will be confidential. Your name and answers will not be shared with your doctor or health plan.

Thank you!

1. Our records show that you are now in MassHealth. Is that right?

- ☐ Yes
- ☐ No

2. Did you hear or see information about MassHealth services to help people quit smoking in any of the following places? (Please answer "yes" or "no" for each one.)

		Yes	No
**a.**	Newspaper	☐	☐
**b.**	Radio	☐	☐
**c.**	Subway, train, or bus	☐	☐
**d.**	Information from MassHealth	☐	☐
**e.**	Doctor’s office or health center	☐	☐
**f.**	Internet website	☐	☐
**g.**	Some other place: ________________ (Please Print.)	☐	☐

3. Using any number between 0 and 10, where 0 is not difficult and 10 is extremely difficult, how difficult do you think it is for **other** people to quit smoking cigarettes?

- ☐ 0 Not Difficult
- ☐ 1
- ☐ 2
- ☐ 3
- ☐ 4
- ☐ 5
- ☐ 6
- ☐ 7
- ☐ 8
- ☐ 9
- ☐ 10 Extremely Difficult

4. Other people's cigarette smoke is sometimes called second hand smoke.

How much does other people's cigarette smoke bother you?

- ☐ A lot
- ☐ Some
- ☐ Only a little
- ☐ Not at all

5. Have you smoked at least 100 cigarettes in your **entire life**?

- ☐ Yes
- ☐ No ➔ **If No, Go to #29 on Page 4**

6. Have you smoked **any** cigarettes during the last 6 months?

- ☐ Yes
- ☐ No ➔ **If No, Go to #29 on Page 4**

Advice from Your Provider

7. During the last 6 months, have you seen a doctor, nurse or other health care professional to get **any kind of care** for yourself?

- ☐ Yes
- ☐ No ➔ **If No, Go to #12**

8. During the last 6 months, did any doctor, nurse or other health care professional advise you to quit smoking?

- ☐ Yes
- ☐ No

9. Counseling services are from a health care professional and can be given on the phone or face-to-face.

In the last 6 months, did you and a doctor, nurse or other health care professional **talk about** counseling to help you quit smoking?

- ☐ Yes
- ☐ No

10. Nicotine replacement can include things like the nicotine patch or nicotine gum.

In the last 6 months, did you and a doctor, nurse or other health care professional **talk about** using nicotine replacement to help you quit smoking?

- ☐ Yes
- ☐ No

11. In the last 6 months, did you and a doctor, nurse or other health care professional **talk about** using medicine such as Chantix or Zyban to help you quit smoking?

- ☐ Yes
- ☐ No

Trying to Quit

12. In your whole life have you ever made a serious attempt to quit smoking?

- ☐ Yes
- ☐ No → **If No, Go to #23**

13. In the last 6 months, did you ever stop smoking cigarettes for more than one day because you were trying to quit?

- ☐ Yes
- ☐ No

14. In the last 6 months, how many times did you try to quit smoking?

- ☐ 0
- ☐ 1
- ☐ 2
- ☐ 3
- ☐ 4
- ☐ 5 or more

15. Thinking back to the last time you tried to quit, how long did you stop smoking?

- ☐ Less than a day
- ☐ 1 to 7 days
- ☐ 8 to 29 days
- ☐ 1 to 3 months
- ☐ More than 3 months

16. As mentioned before, counseling services are from a health care professional and can be given on the phone or face-to-face.

In the last 6 months, did you **try to find** any kind of counseling services to help you quit smoking?

- ☐ Yes
- ☐ No → **If No, Go to #19**

17. In the last 6 months, was it easy or difficult to find someone to give you counseling to quit smoking?

- ☐ Easy → **If Easy, Go to #19**
- ☐ Difficult

18. Why was it difficult to find someone to give you counseling to quit smoking?

____

____

____

____

19. In the last 6 months, did you **get** any kind of counseling services to help you quit smoking?

- ☐ Yes
- ☐ No

20. There are self-help groups like Nicotine Anonymous to help people quit smoking.

In the last 6 months, did you go to a self-help group to help you quit smoking?

- ☐ Yes
- ☐ No

21. The nicotine patch and nicotine gum are examples of nicotine replacement.

In the last 6 months, did you use any kind of nicotine replacement to help you quit smoking?

- ☐ Yes
- ☐ No

22. There are medicines to help people quit smoking like Chantix and Zyban.

In the last 6 months, did you use medicine to help you quit smoking?

- ☐ Yes
- ☐ No

Tobacco Use

23. Do you now smoke cigarettes every day, some days, or not at all?

- ☐ Every day
- ☐ Some days
- ☐ Not at all → **If Not At All, Go to #29**

24. What is the **number** of days you smoked in the last 30 days?

________ # of Days

25. On days that you smoke, how many **cigarettes** do you usually smoke each day?

________ # of Cigarettes

26. On days that you smoke, how soon after you wake up do you usually smoke your first cigarette of the day?

- ☐ Less than 5 minutes
- ☐ 5 to 30 minutes
- ☐ 31 to 60 minutes
- ☐ More than 60 minutes after waking up

27. If you tried to quit smoking, how likely do you think you are to succeed?

- ☐ Not at all likely
- ☐ A little likely
- ☐ Somewhat likely
- ☐ Very likely

28. Do you plan to quit smoking in the next 30 days?

- ☐ Yes
- ☐ No

About You

29. Has a doctor ever told you that you have any of the following conditions? (Please answer "yes" or "no" for each one.)

		Yes	No
**a.**	Asthma	☐	☐
**b.**	Emphysema or COPD (Chronic Obstructive Pulmonary Disease)	☐	☐
**c.**	Lung cancer	☐	☐
**d.**	Colon cancer	☐	☐
**e.**	High blood pressure	☐	☐
**f.**	Heart disease	☐	☐
**g.**	Diabetes	☐	☐

30. Has a doctor or other health professional ever told you that you have depression, anxiety, or some other mental health condition?

☐ Yes
☐ No

31. How many **other** people live with you?

☐ 0 → **If 0, Go to #34**
☐ 1
☐ 2
☐ 3
☐ 4
☐ 5 or more

32. How many children under the age of 12 live with you?

☐ 0
☐ 1
☐ 2
☐ 3
☐ 4
☐ 5 or more

33. How many people who live with you smoke?

☐ 0
☐ 1
☐ 2
☐ 3
☐ 4
☐ 5 or more

34. About how many of your friends smoke cigarettes?

☐ None
☐ A few
☐ Less than half
☐ About half
☐ Most

35. What is your age now?

☐ 18 to 24
☐ 25 to 34
☐ 35 to 44
☐ 45 to 54
☐ 55 to 64
☐ 65 or older

36. Are you male or female?

☐ Male
☐ Female

37. What is the highest grade or level of school you have **completed**?

- ☐ 8th grade or less
- ☐ Some high school, but did not graduate
- ☐ High school graduate or GED
- ☐ Some college or 2-year degree
- ☐ 4-year college degree
- ☐ More than 4-year college degree

38. Are you of Hispanic or Latino origin or descent?

- ☐ Yes, Hispanic or Latino
- ☐ No, not Hispanic or Latino

39. What is your race? (Please mark one or more.)

- ☐ White
- ☐ Black or African-American
- ☐ Asian
- ☐ Native Hawaiian or other Pacific Islander
- ☐ American Indian or Alaska Native
- ☐ Other (Please Print.)

____

40. What language do you **mainly** speak at home?

- ☐ English
- ☐ Cambodian
- ☐ Chinese
- ☐ Haitian / Creole
- ☐ Laotian
- ☐ Portuguese
- ☐ Russian
- ☐ Spanish
- ☐ Vietnamese
- ☐ French
- ☐ Arabic
- ☐ Other (Please Print.) ______________

41. Did someone help you complete this survey?

- ☐ Yes → **If Yes, Go to #42.**
- ☐ No → **If No, Please return the survey in the postage-paid envelope**.

42. How did that person help you? (Please mark one or more.)

- ☐ Read the questions to me
- ☐ Wrote down the answers I gave
- ☐ Answered the questions for me
- ☐ Translated the questions into my language
- ☐ Helped in some other way (Please print.)

____

THANK YOU!

Please return the completed survey in the postage-paid envelope to:

The Center for Survey Research
100 Morrissey Boulevard
Boston, MA 02125
