# Supplementary material for: Massachusetts Medicaid members that smoked in 2008: Characteristics associated with smoking status in 2014
Source: PLoS One. 2017 Oct 12;12(10):e0186144. doi: 10.1371/journal.pone.0186144 (PMC5638442; doi:10.1371/journal.pone.0186144)
Supplement: S2 File — (PDF) [file pone.0186144.s002.pdf]

MassHealth 2014

Smoking Cessation Survey

There are MassHealth benefits that help people to quit smoking - What's your experience?

REMEMBER...

Your Answers Are Always Important To Us
Whether You Smoke...Or Not!!

Survey Instructions

- Answer <u>all</u> the questions by checking the box to the left of your answer.
- You are sometimes told to skip over some questions in this survey. When this happens you will see an arrow with a note that tells you what question to answer next, like this:

☑ Yes ➔ **If Yes, Go to #1 on Page 1**

☐ No

You may have completed a similar MassHealth survey in the past, so some of these survey questions may look familiar. Please answer them anyway. We're interested in knowing what has changed and what has not changed since we last heard from you about smoking.

- **This survey will only take a few minutes.**
  The goal of this research project is to learn about the experiences of MassHealth members in order to better help people to quit smoking. Please answer the questions with your own health and health care in mind.

- **Your name and your answers will be confidential.**
  It is your decision whether or not to complete this survey. Your decision will not affect your MassHealth benefits and will not be shared with your doctor, your health plan, or MassHealth. Your answers will be kept totally confidential; we follow very strict protocols to protect your answers and your personal information.

- **Please return the survey in the postage paid envelope provided**.

Thanks for your help!

If you have any questions about this survey please call:

The Office for Survey Research, UMass Medical School, at 1-888-368-7157.

START HERE:

1. MassHealth records show that you are now in MassHealth. Is that right?

- ☐ Yes
- ☐ No

2. Have you smoked at least 100 cigarettes in your entire life?

- ☐ Yes
- ☐ No ➔ **If No, Go to #37 on Page 4**

3. How old were you the first time you puffed on a cigarette?

_____ Years Old

Tobacco Use

4. Do you now smoke cigarettes every day, some days, or not at all?

- ☐ Every day
- ☐ Some days
- ☐ Not at all ➔ **If Not At All, Go to #28 on Page 3**

5. What is the **number** of days you smoked in the last 30 days?

_____ Number of Days

6. On days that you smoke, how many cigarettes do you usually smoke a day? (1 pack has 20 cigarettes).

_____ Number of Cigarettes

7. On days that you smoke, how soon after you wake up do you usually smoke your first cigarette of the day?

- ☐ Within 5 minutes
- ☐ 6 to 30 minutes
- ☐ 31 to 60 minutes
- ☐ More than 60 minutes after waking up

8. Do you plan to quit smoking in the next 30 days?

- ☐ Yes
- ☐ No

9. How would you rate your interest in trying to quit smoking in the next 6 months?

- ☐ Not at all interested in quitting
- ☐ Somewhat interested in quitting
- ☐ Very interested in quitting

10. If you decided to stop smoking completely, how likely do you think you would be to succeed?

- ☐ Very unlikely
- ☐ Somewhat unlikely
- ☐ Somewhat likely
- ☐ Very likely

11. Using any number between 0 and10, where 0 is not difficult and 10 is extremely difficult, how difficult do you think it is for **other** people to quit smoking cigarettes?

- ☐ 0 - Not at all Difficult
- ☐ 1
- ☐ 2
- ☐ 3
- ☐ 4
- ☐ 5
- ☐ 6
- ☐ 7
- ☐ 8
- ☐ 9
- ☐ 10 - Extremely Difficult

Attempts to Quit

12. Have you ever stopped smoking cigarettes for **more than one day** because you were trying to quit?

- ☐ Yes
- ☐ No ➔ **If No, Go to #37 on Page 4**

13. In the last 12 months, how many times did you try to quit smoking?

- ☐ 0 ➔ **If 0, Go to #15**
- ☐ 1
- ☐ 2
- ☐ 3
- ☐ 4
- ☐ 5 or more

14. Thinking back to the most recent time you tried to quit in the last 12 months, how long did you stop smoking?

- ☐ Less than a day
- ☐ 1 to 6 days
- ☐ 1 week to less than a month
- ☐ 1 month to less than 3 months
- ☐ 3 months to less than 6 months
- ☐ 6 months or more

Getting Help to Quit

We are interested in whether you have used medication or other treatments to help you quit smoking.

15. Nicotine replacement can include things like the nicotine patch or nicotine gum. Have you ever used any kind of nicotine replacement to help you quit smoking?

- ☐ Yes
- ☐ No ➔ **If No, Go to #17**

16. How helpful was nicotine replacement in your attempt to quit smoking?

- ☐ Very helpful
- ☐ Somewhat helpful
- ☐ Not helpful at all

17. Do you think you would use nicotine replacement if you tried to quit again?

- ☐ Yes ➔ **If Yes, Go to #19**
- ☐ No

18. For each statement below, please answer yes or no to indicate if it is a reason why you would not use nicotine replacement.

		Yes	No
**a.**	It is too expensive	☐	☐
**b.**	I think other quit methods will work better for me	☐	☐
**c.**	It will not be effective for me	☐	☐
**d.**	It will affect my physical or mental health in a negative way	☐	☐
**e.**	My doctor recommended against it	☐	☐
**f.**	Some other reason<br>Write in reason: ____________________	☐	☐

19. There is medication to help people quit smoking, such as Chantix or Zyban or Welbutrin. Have you ever used medication to help you quit smoking?

- ☐ Yes
- ☐ No ➔ **If No, Go to #21**

20. How helpful was medication in your attempt to quit smoking?

- ☐ Very helpful
- ☐ Somewhat helpful
- ☐ Not helpful at all

21. Do you think you would use medication if you tried to quit again?

- ☐ Yes ➔ **If Yes, Go to #23 on the next page**
- ☐ No

22. For each statement below, please answer yes or no to indicate if it is a reason why you would not use medication.

		Yes	No
a.	It is too expensive	☐	☐
b.	I think other quit methods will work better for me	☐	☐
c.	It will not be effective for me	☐	☐
d.	It will affect my physical or mental health in a negative way	☐	☐
e.	My doctor recommended against it	☐	☐
f.	Some other reason<br>Write in reason: ______________________	☐	☐

Some people use counseling services to help them quit smoking. Counseling services from a health care professional can be given on the phone or face-to-face.

23. Have you ever used counseling to help you quit smoking?

☐ Yes
☐ No ➔ **If No, Go to #25**

24. How helpful was the counseling in your attempt to quit smoking?

☐ Very helpful
☐ Somewhat helpful
☐ Not helpful at all

25. Do you think you would use counseling if you tried to quit again?

☐ Yes ➔ **If Yes, Go to #27**
☐ No

26. For each statement below, please answer yes or no to indicate if it is a reason why you would not use counseling.

		Yes	No
a.	I will not be able find someone to give me counseling	☐	☐
b.	It is too expensive	☐	☐
c.	I think other quit methods will work better for me	☐	☐
d.	It will not be effective for me	☐	☐
e.	It will affect my physical or mental health in a negative way	☐	☐
f.	Some other reason<br>Write in reason: ______________________	☐	☐

27. What has **helped you the most** in your attempts to quit?

____

____

____

____

➔ **After #27, Go to #37 on the next page**

Have Quit

28. How long has it been since you stopped smoking?

☐ Less than 1 month
☐ 1 month to less than 3 months
☐ 3 months to less than 6 months
☐ 6 months to less than 12 months
☐ 1 year to less than 3 years
☐ 3 years to less than 5 years
☐ 5 years or more

29. Using any number between 0 and 10, where 0 is not difficult and 10 is extremely difficult, how difficult was it is for **you** to quit smoking cigarettes?

- ☐ 0 – Not at all Difficult
- ☐ 1
- ☐ 2
- ☐ 3
- ☐ 4
- ☐ 5
- ☐ 6
- ☐ 7
- ☐ 8
- ☐ 9
- ☐ 10 - Extremely Difficult

We are interested in whether you have used medication or other treatments to help you quit smoking.

30. Nicotine replacement can include things like the nicotine patch or nicotine gum. During the most recent time you quit, did you use any kind of nicotine replacement to help you quit smoking?

- ☐ Yes
- ☐ No ➔ **If No, Go to #32**

31. How helpful was nicotine replacement in your attempt to quit smoking?

- ☐ Very helpful
- ☐ Somewhat helpful
- ☐ Not helpful at all

32. There is medication to help people quit smoking, such as Chantix or Zyban or Welbutrin. During the most recent time you quit, did you use medication to help you quit smoking?

- ☐ Yes
- ☐ No ➔ **If No, Go to #34**

33. How helpful was medication in your attempt to quit smoking?

- ☐ Very helpful
- ☐ Somewhat helpful
- ☐ Not helpful at all

Some people use counseling services to help them quit smoking. Counseling services from a health care professional can be given on the phone or face-to-face.

34. During the most recent time you quit, did you use counseling to help you quit smoking?

- ☐ Yes
- ☐ No ➔ **If No, Go to #36**

35. How helpful was the counseling in your attempt to quit smoking?

- ☐ Very helpful
- ☐ Somewhat helpful
- ☐ Not helpful at all

36. During the most recent time you quit, what **helped you the most** to quit?

____

____

____

____

Electronic Cigarettes

Electronic cigarettes are becoming more common. An electronic cigarette (or e-cigarette) is a battery-powered device which simulates tobacco smoking.

37. In the past 30 days, have you used an e-cigarette (also known as a vapor cigarette), such as NJoy, V2, or Blu?

- ☐ Yes
- ☐ No ➔ **If No, Go to #40 on the next page**

38. How many days of the last 30 days did you use an e-cigarette?

_______ Number of Days

39. For each statement below, please answer yes or no to indicate if it is a reason why you use e-cigarettes.

		Yes	No
**a.**	To quit other tobacco	☐	☐
**b.**	To replace other tobacco	☐	☐
**c.**	To cut down on other tobacco	☐	☐
**d.**	To use in places where other tobacco is not allowed	☐	☐
**e.**	Curiosity – just to try it	☐	☐
**f.**	Some other reason<br>Write in reason: ______________________	☐	☐

About You

40. How many people who live with you smoke?

- ☐ 0
- ☐ 1
- ☐ 2
- ☐ 3
- ☐ 4
- ☐ 5 or more

41. About how many of your friends smoke cigarettes?

- ☐ None
- ☐ A few
- ☐ Less than half
- ☐ About half
- ☐ Most

42. What is your age now?

- ☐ 18 to 24
- ☐ 25 to 34
- ☐ 35 to 44
- ☐ 45 to 54
- ☐ 55 to 64
- ☐ 65 or older

43. How do you identify your gender?

- ☐ Female
- ☐ Male
- ☐ Transgender

44. What is the highest grade or level of school you have **completed**?

- ☐ 8th grade or less
- ☐ Some high school, but did not graduate
- ☐ High school graduate or GED
- ☐ Some college or 2-year degree
- ☐ 4-year college degree
- ☐ More than 4-year college degree

45. Are you currently working at a job for pay?

- ☐ Yes
- ☐ No

46. Are you of Hispanic or Latino origin or descent?

- ☐ Yes, Hispanic or Latino
- ☐ No, not Hispanic or Latino

47. What is your race? (Please mark one or more.)

- ☐ White
- ☐ Black or African-American
- ☐ Asian
- ☐ Native Hawaiian or other Pacific Islander
- ☐ American Indian or Alaska Native
- ☐ Other (Write in) ____________________

48. What language do you **mainly** speak at home?

- ☐ English
- ☐ American Sign Language
- ☐ Arabic
- ☐ Cambodian
- ☐ Chinese
- ☐ Haitian / Creole
- ☐ Laotian
- ☐ Portuguese
- ☐ Russian
- ☐ Spanish
- ☐ Vietnamese
- ☐ Other (Write in) ____________________

49. Has a doctor or other health professional ever told you that you have any of the following conditions? (Please answer 'Yes' or 'No' for each one.)

		Yes	No
a.	Asthma	☐	☐
b.	Emphysema or COPD (Chronic Obstructive Pulmonary Disease)	☐	☐
c.	Lung cancer	☐	☐
d.	Colon cancer	☐	☐
e.	High blood pressure	☐	☐
f.	Heart disease	☐	☐
g.	Diabetes	☐	☐

50. Has a doctor or other health professional ever told you that you have depression, anxiety, or some other mental health condition?

- ☐ Yes
- ☐ No

51. During the past 12 months, have you seen a doctor, nurse or other health care professional to get **any kind of care** for yourself?

- ☐ Yes
- ☐ No ➔ **If No, Go to #53**

52. During the last 12 months, did any doctor, nurse or other health care professional advise you to quit smoking?

- ☐ Yes
- ☐ No

53. Did someone help you complete this survey?

- ☐ Yes
- ☐ No ➔ **If No, Go to END**

54. How did that person help you? (Please check one or more.)

- ☐ Read the questions to me
- ☐ Wrote down the answers I gave
- ☐ Answered the questions for me
- ☐ Translated the questions into my language
- ☐ Helped in some other way (Write in)

____

____

END

Thank you! Please return the completed survey in the postage-paid envelope to:

The Office of Survey Research
University of Massachusetts Medical School
Center for Health Policy and Research
333 South Street
Shrewsbury, MA 01545
